# The Association of Oxygenation, Carbon Dioxide Removal, and Mechanical Ventilation Practices on Survival During Venoarterial Extracorporeal Membrane Oxygenation

**DOI:** 10.3389/fmed.2021.756280

**Published:** 2021-11-18

**Authors:** Angelo Justus, Aidan Burrell, Chris Anstey, George Cornmell, Daniel Brodie, Kiran Shekar

**Affiliations:** ^1^Adult Intensive Care, Sunshine Coast University Hospital, Sunshine Coast, QLD, Australia; ^2^Australian and New Zealand Intensive Care-Research Centre, School of Public Health and Preventive Medicine, Monash University, Melbourne, VIC, Australia; ^3^Department of Intensive Care, The Alfred Hospital, Melbourne, VIC, Australia; ^4^School of Medicine, Griffith University, Brisbane, QLD, Australia; ^5^Faculty of Medicine, University of Queensland, Brisbane, QLD, Australia; ^6^Adult Intensive Care Services, The Prince Charles Hospital, Brisbane, QLD, Australia; ^7^Department of Medicine, Columbia University College of Physicians and Surgeons, New York, NY, United States; ^8^Center for Acute Respiratory Failure, New York-Presbyterian Hospital, New York, NY, United States; ^9^Institute of Health and Biomedical Innovation, Queensland University of Technology, Brisbane, QLD, Australia

**Keywords:** extracorporeal membrane oxygenation, venoarterial extracorporeal membrane oxygenation, extracorporeal circulation, hyperoxemia, oxygenation, mechanical ventilation, carbon dioxide removal

## Abstract

**Introduction:** Oxygenation and carbon dioxide removal during venoarterial extracorporeal membrane oxygenation (VA ECMO) depend on a complex interplay of ECMO blood and gas flows, native lung and cardiac function as well as the mechanical ventilation strategy applied.

**Objective:** To determine the association of oxygenation, carbon dioxide removal, and mechanical ventilation practices with in-hospital mortality in patients who received VA ECMO.

**Methods:** Single center, retrospective cohort study. All consecutive patients who received VA ECMO in a tertiary ECMO referral center over a 5-year period were included. Data on demographics, ECMO and ventilator support details, and blood gas parameters for the duration of ECMO were collected. A multivariable logistic time-series regression model with in-hospital mortality as the primary outcome variable was used to analyse the data with significant factors at the univariate level entered into the multivariable regression model.

**Results:** Overall, 52 patients underwent VA ECMO: 26/52 (50%) survived to hospital discharge. The median PaO_2_ for the duration of ECMO support was 146 mmHg [IQR 131–188] and PaCO_2_ was 37.2 mmHg [IQR 35.3, 39.9]. Patients who survived to hospital discharge had a significantly lower median PaO_2_ (117 [98, 140] vs. 154 [105, 212] mmHg, *P* = 0.04) and higher median PaCO_2_ (38.3 [36.1, 41.1] vs. 36.3 [34.5, 37.8] mmHg, *p* = 0.03). Survivors also had significantly lower median VA ECMO blood flow rate (EBFR, 3.6 [3.3, 4.2] vs. 4.3 [3.8, 5.2] L/min, *p* = < 0.001) and greater measured minute ventilation (7.04 [5.63, 8.35] vs. 5.32 [4.43, 6.83] L/min, *p* = 0.01). EBFR, PaO_2_, PaCO_2_, and minute ventilation, however, were not independently associated with death in a multivariable analysis.

**Conclusion:** This exploratory analysis in a small group of VA ECMO supported patients demonstrated that hyperoxemia was common during VA ECMO but was not independently associated with increased mortality. Survivors also received lower EBFR and had greater minute ventilation, but this was also not independently associated with survival. These findings highlight that interactions between EBFR, PaO_2_, and native lung ventilation may be more relevant than their individual association with survival. Further research is indicated to determine the optimal ECMO and ventilator settings on outcomes in VA ECMO.

## Introduction

Venoarterial extracorporeal membrane oxygenation (VA ECMO) is a form of life support that provides gas exchange and circulatory support for severe cardiac failure or for refractory cardiac arrest (1, 2). Significant advances in extracorporeal technology and accumulating data have led to more widespread use of ECMO (3). Despite the increasing use of VA ECMO to support patients with cardiac failure, many questions remain unanswered. One such question relates to gas exchange targets during VA ECMO and another on how best to apply mechanical ventilation during VA ECMO support. Currently, targets for the partial pressure of arterial oxygen (PaO_2_), and carbon dioxide (PaCO_2_) and for mechanical ventilation settings are guided by local practices and there are no unified, evidence-based guidelines to direct practice.

PaO_2_ and PaCO_2_ in patients undergoing VA ECMO support is dependent on complex interactions between the ECMO circuit, native lung/heart functions, and ventilatory support being provided. ECMO circuit factors include the fraction on delivered oxygen (FdO_2_) in the sweep gas, ECMO blood flow rate (EBFR) ECMO sweep gas flow rate (SGFR), and oxygenator efficiency. Pertinent patient factors include native cardiac output, native lung function, carbon dioxide production (VCO_2_), and oxygen consumption (VO_2_). Mechanical ventilation settings such asset FiO_2_, respiratory rate, and positive end-expiratory pressure (PEEP) also play a key role. The occurrence of hyperoxemia (PaO_2_ >101–300 mmHg) and hypocarbia (PaCO_2_ <30 mmHg) in VA ECMO patients are both common (4, 5), despite unclear impact on patient outcomes, due lack of evidence to guide practice, and concerns amongst clinicians due to the potential risk of inadvertent differential oxygenation in patients with VA ECMO and dual circulations (3, 6, 7).

Excessive blood oxygenation (hyperoxemia) may have deleterious consequences in this high-risk group of patients receiving VA ECMO. Reactive oxygen species (ROS) are by-products of hyperoxemia and tissue hyperoxia, which are thought to result in vasoconstriction, cell damage, inflammation, and cell death (8–11). ROS have been postulated to be of major significance in tissue damage, organ dysfunction, and increased patient morbidity and mortality (9, 12–14). The harmful effects of hyperoxemia on ECMO may be dependent on the underlying condition, duration, and degree of the hyperoxemic exposure (5, 13, 15–17). A lack of universal definition for hyperoxemia, lack of high-quality evidence, and lack of oxygen weaning guidelines have resulted in patients being exposed to prolonged periods of hyperoxemia on ECMO.

Similarly, there is emerging evidence suggesting that a higher PaCO_2_ may be protective in critically ill patients (18–21). CO_2_ not only inhibits generation of ROS (20) by cells, the main physiological effect of increased CO_2_ in patients might be due to rightward shift of the oxyhemoglobin dissociation curve, resulting in improved unloading of oxygen and better tissue oxygenation (21, 22). Equally, in patients receiving venovenous ECMO for respiratory failure, a large relative decrease in PaCO_2_ in the first 24 h after ECMO initiation may be associated with an increased incidence of neurological complications (23–26). However, such an association hasn't been demonstrated in patients receiving VA ECMO support. Similarly, how to ventilate a patient's native lung in this cohort of patients is also not guided by evidence. Lung injury in patients receiving VA ECMO support is not uncommon due to pulmonary oedema from heart failure and fluid overload as well as from ventilator-associated pneumonia.

Therefore, this single center study aimed to investigate the effects of oxygenation, carbon dioxide removal, and native lung ventilation practices on survival in patients receiving VA ECMO support.

## Methods

### Design and Setting

This was a single center retrospective cohort study conducted in a tertiary ECMO referral center in Queensland, Australia. The Study hospital offers heart and lung transplantation service for a population of more than 5 million in the state of Queensland, Australia. The hospital also provides ECMO retrieval service for hospitals across Queensland. The intensive care unit (ICU) has more than 1,800 admissions and ~30–35 ECMO cases each year. Ethics approval was obtained (HREC/18/QPCH/199) prior to commencement of the study.

### Population

All consecutive patients supported by VA ECMO from 1st January 2012 through December 2017 were included in the study. Patients who received venovenous ECMO or were <18 years old were excluded.

### Daily ECMO Management

Patients on VA ECMO support were managed by intensive care specialists trained in ECMO and most practices are protocolized as per the intensive care unit guidelines. ECMO blood flows were typically titrated to facilitate native cardiac ejection aiming for a pulse pressure of at least 20 mmHg, further aided by inotropic therapy and an intra-aortic balloon pump. The PEEP was usually set at a moderate level (10–15 Cm H_2_O) especially in patients who are at risk of left ventricular distension. Anticoagulation was achieved with an unfractionated heparin infusion targeting an activated partial thromboplastin time of at least 60 s with further increases in intensity of anticoagulation in patients with left ventricular distension. The perfusion strategy was revised to a temporary biventricular assist device configuration with oxygenator in 3 patients and an isolated left ventricular assist device with oxygenator in one patient owing to left ventricular distention. Configuration was changed to venovenous in 8 patients with poor pulmonary reserve upon sufficient cardiac recovery and additional venous return was provided in 2 patients who developed severe differential oxygenation during VA ECMO.

Irrespective of the ECMO configuration (peripheral or central), arterial blood gases (ABG) were taken from the right radial or right brachial arterial line. ABGs were done at the discretion of treating clinicians. All the ABGs done on a single day were used to calculate daily mean PaO_2_, PCO_2_, hemoglobin (Hb), and oxygen saturation (SaO_2_). Daily mean oxygen content (CaO_2_) was calculated from ABGs using the arterial oxygen content equation: CaO_2_ = (1.34 ^*^ Hb ^*^ SaO_2_) + (0.0031 ^*^ PaO_2_).

ECMO parameters, including EBFR, FdO_2_, SGFR, and ventilator parameters (tidal volume, ventilator FiO_2_, minute volume, peak airway pressure, PEEP, and respiratory rate) were recorded every hour in our electronic medical record. Daily mean and standard deviation (SD) or median and interquartile range (IQR) for VA ECMO and ventilator parameters were calculated from these recordings.

Ventilator management and ECMO support was at the discretion of the treating intensivists. ECMO FdO2, ECMO SGFR, ventilator FiO_2_, and ventilator supports were adjusted daily by intensivists based on the patients' clinical status, bedside assessment of cardiac and lung function and results of ABGs, targeting normocarbia (PaCO_2_ 35–45 mmHg), a PaO_2_ between 60 and 100 mmHg and mean arterial pressure (MAP) between 65 and 70 mmHg. Hyperoxia was defined as PaO_2_ >100 mmHg for the purposes of this study.

Patients were assessed daily using weaning guidelines for liberation from VA ECMO support, which involves multidisciplinary team discussions, serial echocardiography, and assessments of patient clinical state.

### Data Collection

Patients were identified using a prospectively collected hospital ECMO database maintained by the ICU, and the extracorporeal life support organization (ELSO) registry. All collected data were cross checked using the hospital electronic medical record.

The following data were collected: baseline demographics and illness severity scores [sequential organ failure assessment (SOFA) and acute physiology, age, chronic health evaluation 3 (APACHE 3)], diagnostic group, cannulation configuration (peripheral vs. central), ECMO specific parameters, such as EBFR, ECMO SGFR, FdO_2_, ventilator parameters such as ventilator FiO_2_, PEEP, peak airway pressure, tidal volume, minute ventilation, and respiratory rate. The following patient outcome data were also collected: ICU and hospital mortality, ICU and hospital length of stay (LOS), duration of ECMO support, and duration of mechanical ventilation.

### Statistical Analysis

The data constituted a time-series and were analyzed and summarized according to distribution. Normally distributed data were analyzed with a two-tailed unpaired *t*-test and summarized using mean and SD, whereas data with a non-normal distribution were analyzed with a two-tailed unpaired Wilcoxon rank-sum test and summarized using median and IQR, dichotomous data were analyzed with Fisher's exact test and summarized using *n*/*N* (%). A multivariable logistic time-series regression model with hospital outcome as the outcome variable was used to analyse the data with significant factors at the univariate level entered into the multivariable regression model. Initial predictor variables included APACHE III score, FdO_2_, minute volume, peak airway pressure, PEEP, hemoglobin, PaO_2_, PaCO_2_, and days on ECMO.

In the first instance, a fully saturated model was used with sequential deletion of non-significant predictors until a final model was reached. Likelihood ratio testing between iterations was used to validate predictor exclusion. Significant findings were reported using the odds ratio and its 95% confidence interval. A significance level of *P* < 0.05 was used throughout and all analyses were performed with STATA (version 15.0).

## Results

Fifty-two patients were included in the final analysis. Table 1 shows the baseline characteristics, ventilator settings, ECMO support details, and outcomes of the patients receiving VA ECMO. Among the 52 patients, 19 (36.5%) patients received VA ECMO support following cardiac surgical procedures and the indications for surgery were coronary artery bypass grafting, valve replacement, heart transplant, and lung transplant. Non-surgical indications for VA ECMO support were heart failure due to myocardial infarction, cardiomyopathies, and myocarditis (Table 2). Peripheral VA ECMO configuration was used in 35 (67%) patients and 17 (33%) patients had central VA ECMO configuration.

**Table 1:** Baseline demography, ECMO support, and ventilation characteristics.

**Variable**	**Survivors (*n* = 26)**	**Dead (*n* = 26)**	***p*-value**
**Demographics**			
Age—mean (SD), years	44.9 (15.9)	51.9 (15.8)	0.12
Male sex—no./total no. (%)	18/26 (69%)	18/26 (69%)	1
Calendar days on VA ECMO—median [IQR], days	7.0 [5.8, 9.3]	4.5 [3.0, 7.3]	0.02
Duration of mechanical ventilation—median [IQR], days	20 [12, 34]	8[4, 17]	0.003
ICU length of stay—median [IQR], days	25 [15, 38]	9 [4, 19]	<0.001
Hospital length of stay—median [IQR], days	41 [24, 65]	9 [5, 24]	<0.001
APACHE III—median [IQR]	78 [28, 107]	99 [62, 135]	0.04
SOFA score on day 1—median [IQR]	10 [8, 12]	13 [8, 16]	0.11
**VA ECMO support details**			
Blood flow rate—median [IQR], L/min	3.6 [3.3, 4.2]	4.3 [3.8, 5.2]	<0.001
Sweep gas flow rate—median [IQR], L/min	4.0 [2.0, 5.0]	4.1 [3.3, 6.4]	0.22
FdO_2_–median [IQR], %	71.7 [61.8, 82.2]	78.0 [70.0, 86.7]	0.31
**Ventilator support details**			
FiO_2_ (ventilator)—median [IQR], %	45.0 [42.3, 52.3]	47.2 [42.7, 60.7]	0.47
Respiratory rate/min—median [IQR]	16.7 [13.1, 19.8]	14.7 [12.2, 20.1]	0.56
Tidal volume—median [IQR], ml	416 [356, 515]	385 [287, 432]	0.06
Minute ventilation—median [IQR], L/min	7.04 [5.63, 8.35]	5.32 [4.43, 6.83]	0.01
Peak airway pressure—median [IQR], CmH_2_O	23.4 [21.6, 25.1]	24.3 [21.8, 29.0]	0.24
PEEP—median [IQR], CmH_2_O	10.0 [8.0, 10.8]	10.4 [8.1, 12.4]	0.35

*SD, standard deviation; IQR, inter quartile range; VA ECMO, venoarterial extracorporeal membrane oxygenation; APACHE, Acute Physiology and Chronic Health Evaluation; SOFA, Sequential Organ Failure Assessment score, FiO_2_, Fraction of Inspired Oxygen; FdO_2_, fraction of delivered oxygen via ECMO; PEEP, Positive End Expiratory Pressure*.

**Table 2:** Indications for ECMO.

**Medical**	33 (63%)
Chronic decompensated cardiomyopathy	5
Arrhythmia	1
Cardiogenic shock	17
Myopericarditis	1
Endocarditis	1
Angina	1
Septic shock	2
No diagnosis	1
**Surgical**	19 (36%)
Post-infarct ventricular septal defect	1
Thoracic aortic aneurism with dissection	2
Coronary artery bypass grafting and valve	5
Respiratory surgery	1
Valvular surgery	1
Vascular surgery	1
Heart transplant	2
Coronary artery bypass grafting	2
Lung transplant	2
Pulmonary endarterectomy	2
**ECMO assisted cardiopulmonary resuscitation**	11 (21%)
Medical	8
Surgical	3


Thirty-five (67%) patients were successfully liberated from ECMO support with 27 (52%) patients surviving ICU discharge and 26 (50%) patients surviving to hospital discharge. Age, Sex, SOFA score on day 1 of ICU admission, ECMO SGFR, Fdo_2_, and FiO_2_, respiratory rate, tidal volume, peak airway pressure, PEEP, mean hemoglobin concentration, and oxygen content were similar in both hospital survivors and hospital non-survivors. Patients who died in the hospital had higher APACHE III score (78 vs. 99, *p* = 0.04). Duration of ECMO support was longer in the patients who survived to hospital discharge.

**Figure 1:**
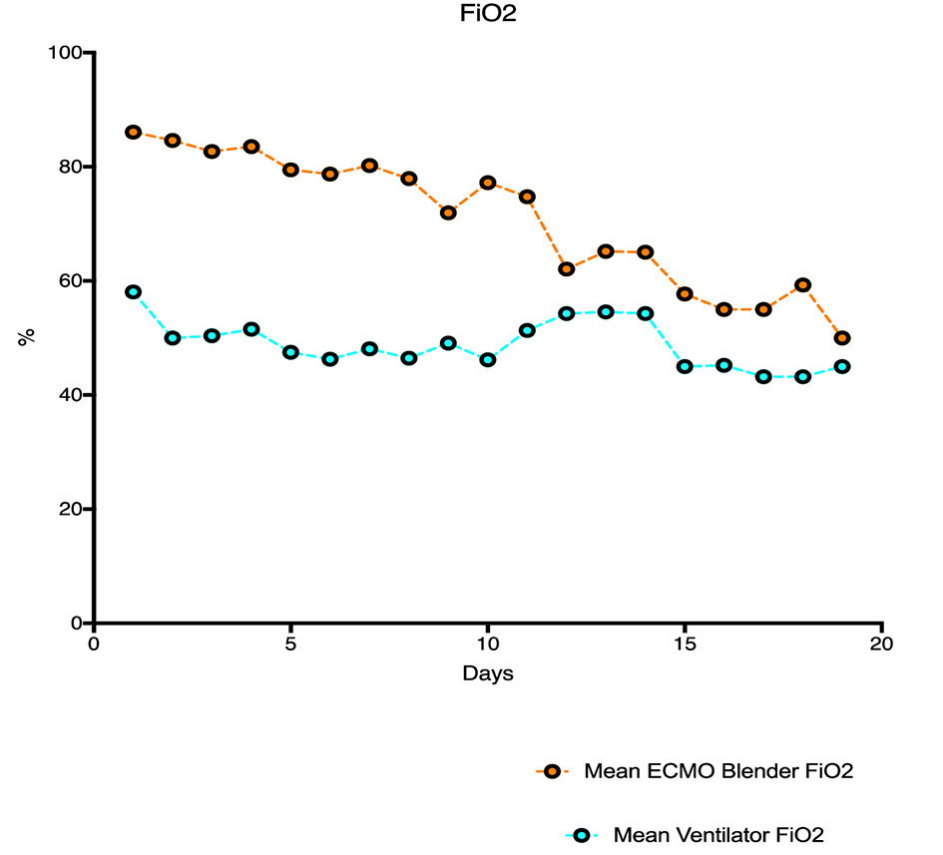
Mean ECMO blender FiO_2_ and mean ventilator FiO_2_ for the duration of ECMO support.

**Figure 2:**
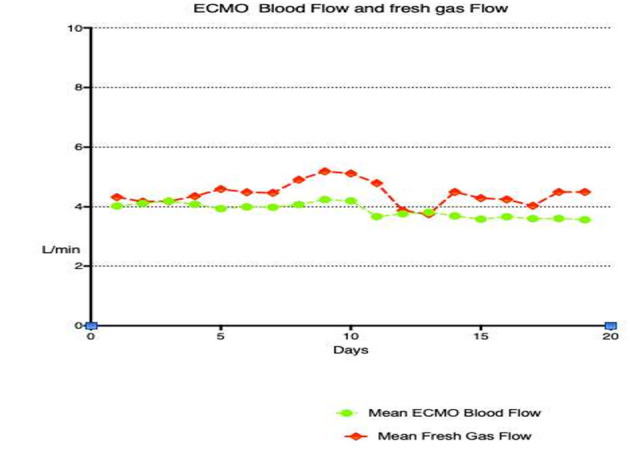
ECMO sweep gas flow rate and ECMO blood flow rate for the duration of VA ECMO support.

**Figure 3:**
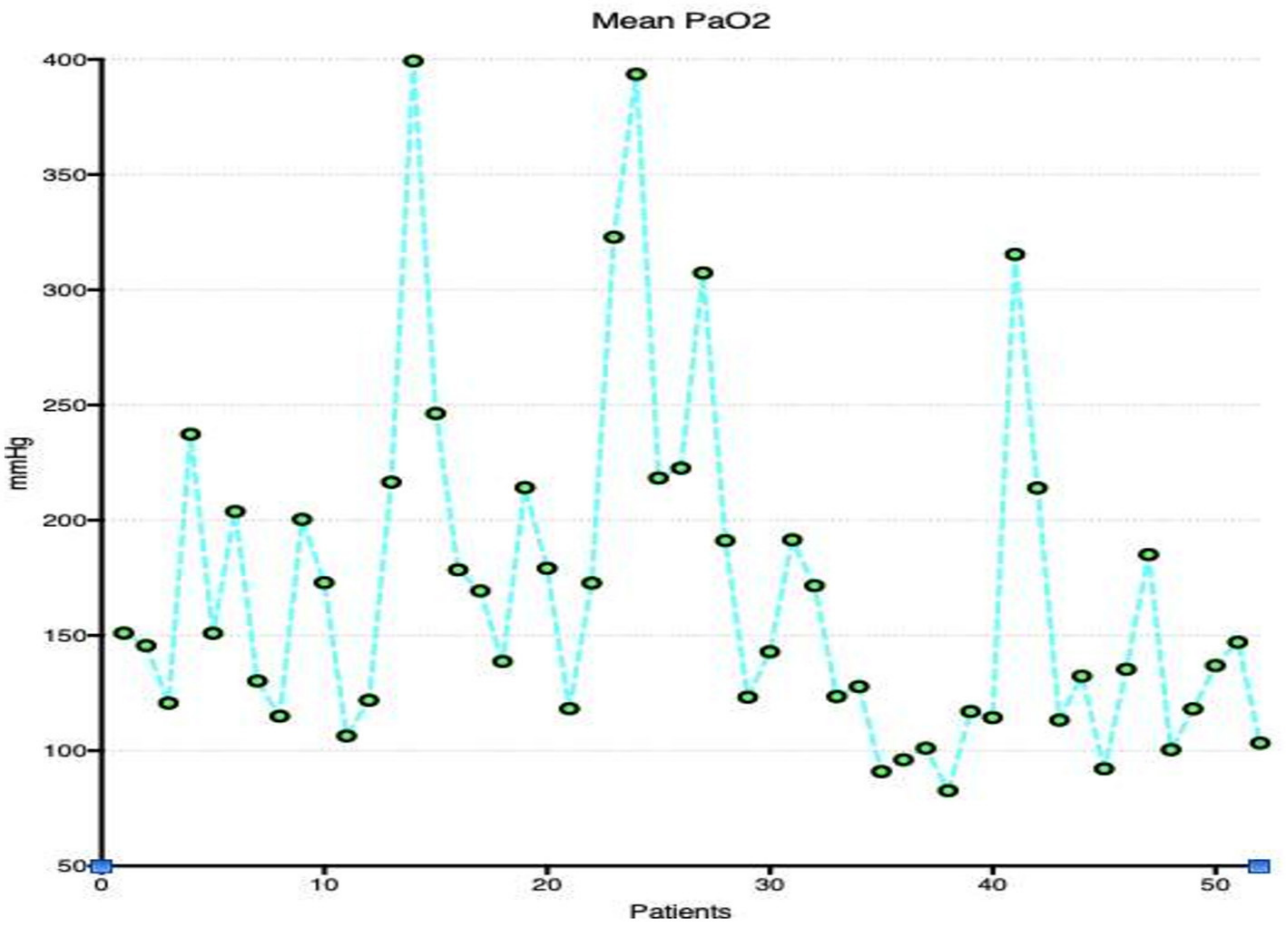
Mean PaO_2_ for individual patients during their entire duration of ECMO support.

Oxygenation and carbon dioxide removal parameters are summarized in Table 3. Figure 1 demonstrates mean daily FdO_2_ and ventilator FiO_2_ for the duration of ECMO support in the study population. FdO_2_ declined significantly over time, however, FiO_2_ remained relatively constant for the duration of VA ECMO support. Figure 2 shows EBFR and SGFR remained relatively constant during the entire duration of VA ECMO support. Figure 3 shows mean PaO_2_ for individual patients during their entire duration of ECMO support. There was a large variability between patients, with mean PaO_2_ ranging from 82.7 to 400 mmHg. Two patients who exhibited a mean Pao_2_ >400 mmHg had an outlier effect owing to their short ECMO runs. One died at 27 h and the other was liberated from ECMO at 42 h.

**Table 3:** Oxygenation and carbon dioxide removal parameters.

**Variable**	**Survivors (*N* = 26)**	**Dead (*N* = 26)**	***p*-value**
PaO_2_–median [IQR], mmHg	117 [98, 140]	154 [105, 212]	0.04
SaO_2_-median [IQR], %	98.5 [97.4, 99.1]	98.8 [97.4, 99.5]	0.46
PaCO_2_–median [IQR], mmHg	38.3 [36.1, 41.1]	36.3 [34.5, 37.8]	0.03
Hemoglobin—median [IQR], g/L	84.3 [81.1, 88.1]	82.9 [80.1, 85.6]	0.32
Oxygen content—median [IQR], ml/L	112.7 [109.1, 117.7]	112.3 [107.5, 115.8]	0.33

*IQR, inter quartile range; Pao_2_, partial pressure of oxygen; Sao_2_, arterial oxygen saturation; Paco_2_, partial pressure of carbon dioxide*.

**Figure 4:**
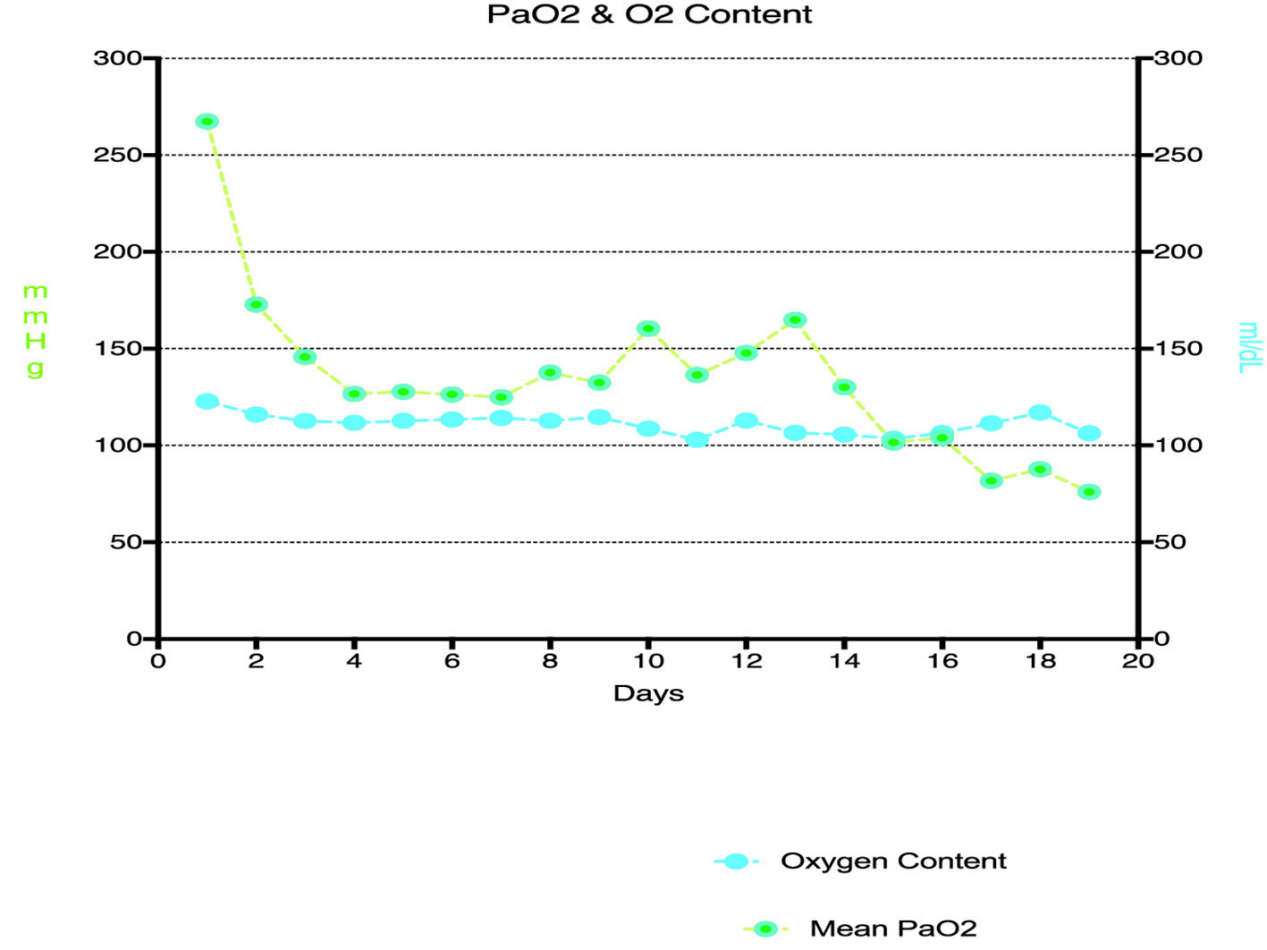
Relationship between PaO_2_ and oxygen content for the duration of VA ECMO support.

**Figure 5:**
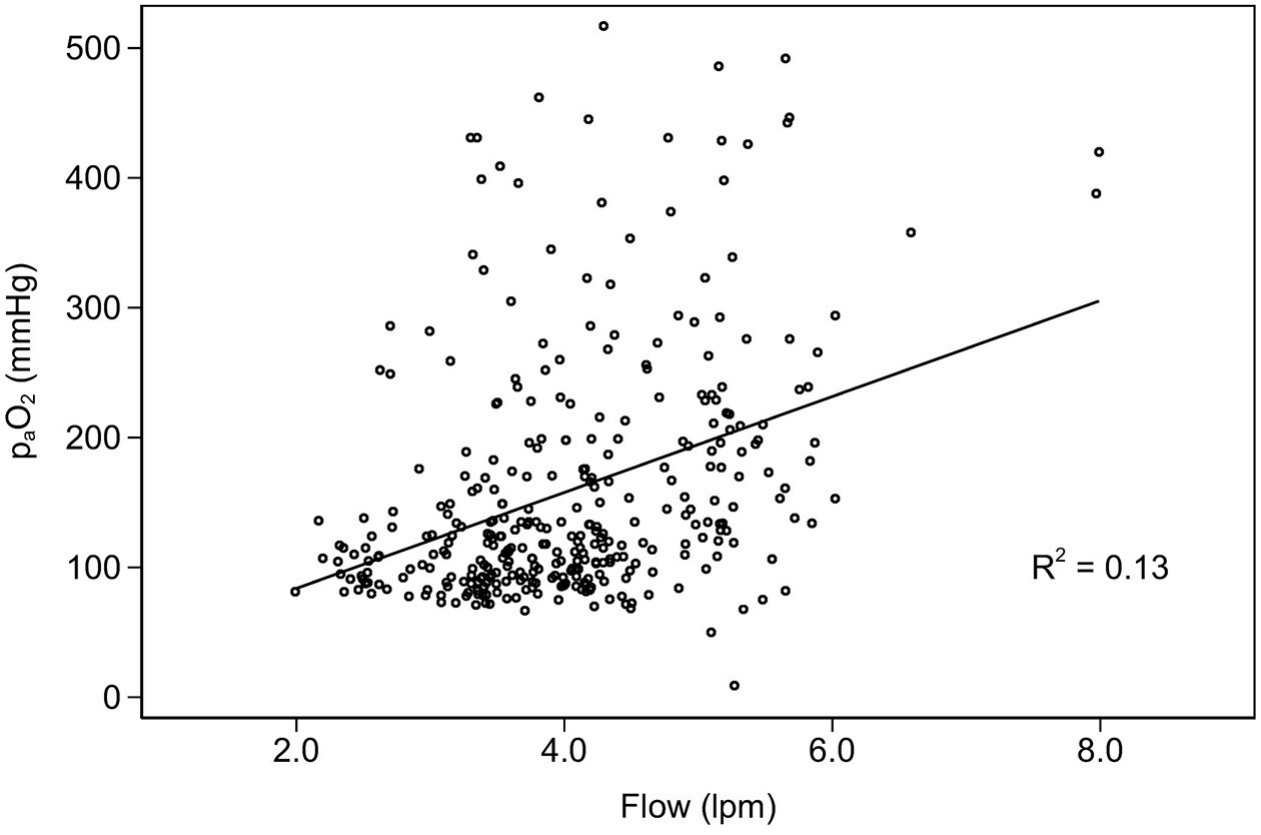
Interaction between ECMO blood flow rate and partial pressure of oxygen.

Figure 4 shows elevated PaO_2_ during VA ECMO support with severe hyperoxemia during early stages after commencement of VA ECMO and the relationship between right radial PaO_2_ and calculated oxygen content in the study population during the study period. Large changes in PaO_2_ contributed to small changes to oxygen content in arterial blood. However, there was poor correlation between EBFR and PaO_2_ (Figure 5).

**Figure 6:**
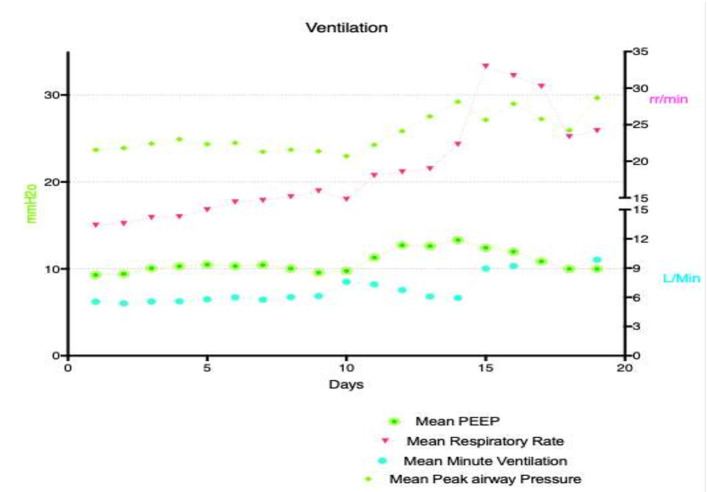
Mechanical ventilation parameters for the duration of ECMO support.

**Figure 7:**
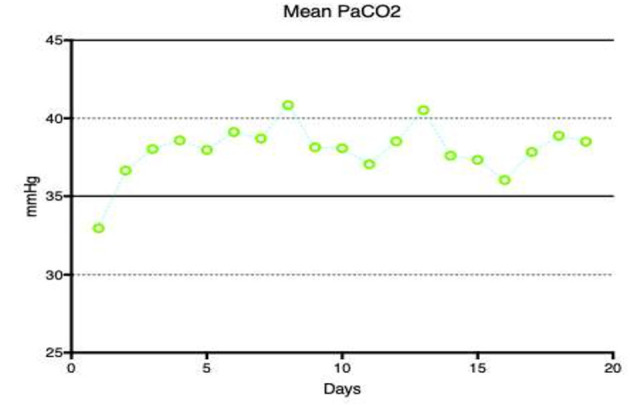
Mean PaCo_2_ for the duration of ECMO support.

Ventilation parameters are demonstrated in Figure 6. PEEP stayed relatively constant, however respiratory rate and minute ventilation progressively increased during the course of VA ECMO support. Figure 7 demonstrates daily mean PaCO_2_ in the study population. Normocarbia was maintained for the duration of VA ECMO support except for the first day after initiation of VA ECMO.

When compared to non-survivors, hospital survivors had a significantly lower EBFR (3.6 vs. 4.3 L/min, *p* = < 0.001) and received significantly higher native lung minute ventilation (7.04 vs. 5.32 L/min, *p* = 0.01) with a trend toward larger tidal volumes (Table 1). Hospital survivors had significantly lower cumulative mean PaO_2_ than the patients who died in the hospital (117 vs. 154 mmHg *p* = 0.04). Hospital survivors also had significantly higher cumulative mean PaCO_2_ (38.3 vs. 36.3 mmHg *p* = 0.03) than hospital non-survivors (Table 3). On multivariable logistic time-series regression analysis, hyperoxemia, PaCO_2_, and minute ventilation were not independently associated with in-hospital mortality (Table 4).

**Table 4:** Multivariable regression analysis.

**Variable**	**Initial model**		**Final model**	
	**OR (95%CI)**	***P*-value**	**OR (95%CI)**	***P*-value**
ECMO days	1.19 (0.98, 1.44)	0.083	1.19 (0.99, 1.44)	0.067
APACHE III	0.99 (0.97, 1.01)	0.235	–	–
Minute ventilation	1.14 (0.87, 1.48)	0.341	–	–
PaO_2_	0.99 (0.98, 1.01)	0.509	–	–
PaCO_2_	1.09 (0.96, 1.25)	0.188	–	–
ECMO blood flow rate	0.53 (0.23, 1.20)	0.125	0.52 (0.25, 1.06)	0.073

*ECMO, extracorporeal membrane oxygenation; APACHE, Acute Physiology and Chronic Health Evaluation; PaO_2_, partial pressure of oxygen; PaCO_2_, partial pressure of carbon dioxide*.

## Discussion

This exploratory analysis in a small cohort of VA ECMO supported patients demonstrated that significant levels of hyperoxemia during ECMO support and that there was significant inter-individual variability in exposure. However, hyperoxemia was not independently associated with an increased in-hospital mortality. In addition, PaCO_2_, EBFR, and minute ventilation were also not independently associated with mortality.

This study examined exposure to hyperoxemia during the entire ECMO run. However, studies looking at prevalence of hyperoxemia in the early stages (1st 48 h) of ECMO runs have consistently shown that hyperoxemia is common in ECMO patients (3, 4). The cause of hyperoxemia in these cohort of patients is likely to be multifactorial, including lack of a clear definition for hyperoxemia. The PaO_2_ threshold at which harm outweighs benefit is unknown and there are no universally agreed upon guidelines in weaning FdO_2_ or ventilator FiO_2_ in this cohort of patients. It is important to remember that PaO_2_ in VA ECMO patients is dependent on complex interactions between multiple factors including native lung function and pulmonary blood flow, native cardiac function, ECMO blood flow and FdO_2_, and ventilator settings including FiO_2_ (27). The factors that influence PaO_2_ in an individual patient receiving VA ECMO may be dynamic and poses a challenge in managing these patients. For example, with recovering native cardiac function in peripheral VA ECMO patients there is a real risk of inadvertent differential oxygenation if the native lung function is poor (6). The risk of differential oxygenation may be one of the barriers in weaning ventilator FiO_2_ or FdO_2_ in this cohort of patients. Advanced monitoring, including continuous SaO_2_ monitoring or cerebral tissue oxygenation monitoring, may be required to identify and treat differential oxygenation (28).

Our study suggests that severe degrees of hyperoxemia (PaO_2_ >200 mmHg) during the early stages of VA ECMO support is also very common. This is consistent with prior studies in ECMO patients (4, 5, 16, 17). VA ECMO patients are critically unwell and reluctance among physicians in making too many early changes may also contribute to severe hyperoxemia during the early stages of VA ECMO support. Our study also shows that, mean PaO_2_ between patients varies widely for the duration of their ECMO support. The heterogeneity in underlying cardiac pathology and consequently different degrees of native cardiac ejection in patients supported with VA ECMO may result in variable individual patient exposures to hyperoxaemia. In addition, different rates and extent of cardiac recovery may also mean the exposure to hyperoxaemia may be highly variable between patients over the course of ECMO. This interindividual variability apart from patient factors also reflects the absence of standardized guidelines for weaning FdO_2_ and ventilator FiO_2_ in this cohort of patients and also shows differences in practice among clinicians. Prevalence of hyperoxemia in VA ECMO patients and potential harm associated with hyperoxemia means, clinicians caring for patients receiving VA ECMO support should be vigilant. Hyperoxemia should prompt clinicians to wean FiO_2_ on the ventilator or ECMO blender based on the patients' native heart function, EBFR, transpulmonary blood flow, native lung function, and type of ECMO configuration (Peripheral vs. Central). In peripheral VA ECMO configuration all care should be taken to prevent differential oxygenation when there is dual circulation. Intensive care units should begin to develop standardized guidelines/approaches in managing hyperoxemia in this cohort of patients, although guiding these practices will be difficult till there is more conclusive data.

Our study finding is in contrast with multiple studies suggesting hyperoxemia is associated with increased mortality in VA ECMO supported patients. Al-Kawaz et al. (17) in a single center study reported that duration and severity of early hyperoxemia were independently associated with poor neurologic outcomes at discharge as well as with mortality. However, Munshi et al. (5) reported potential harm associated with hyperoxemia in venovenous ECMO patients and in those receiving VA ECMO in the context of cardiopulmonary resuscitation. Several possible factors may account for this. The interactions between EBFR, native cardiac ejection, native lung status, Fdo_2_, Fio_2_, and PEEP all determine overall exposure to hyperoxemia. From first principles, patients with poor native cardiac ejection, who are dependent on higher EBFRs are typically at higher risk of hyperoxemia if Fdo_2_ is set at 100%. If Fdo_2_ and EBFR remain unchanged, patients will experience variable degrees of hyperoxemia over the course of the ECMO run based on cardiac recovery and native lung status. It should be noted that, there are conflicting data on optimal blood flow rate settings during VA ECMO. In this study, patients who received lower EBFRs had better outcomes in a univariable analysis but EBFR did not appear to independently affect survival. Thus, severe hyperoxemia may largely be reflective of the degree of cardiac dysfunction and prognosis therefore may be more reliant on cardiac recovery or lack thereof as well as other complications suffered during the ECMO run (27). This may potentially explain the lack of independent associations of either hyperoxemia or EBFR on survival in this cohort.

Similarly, although survivors had significantly higher cumulative PaCO_2_ exposure in a univariable analysis, this did not appear to affect outcome in multivariable analysis. The protective effect of higher PaCO_2_ may be due to improved unloading of oxygen from rightward shift of the oxyhemoglobin dissociation curve (29) resulting in improved tissue oxygenation as well as reduced generation of ROS (20) and as such is a matter for future research. Studies have shown an association between rapid reduction in PaCO_2_ upon ECMO initiation and adverse neurological outcomes, more so in those patients receiving venovenous ECMO support (23) for acute respiratory distress syndrome. PaCO_2_ in patients receiving VA ECMO can be effectively controlled by altering ECMO SGFR and to a lesser degree by altering native lung ventilation. Resting the lung with minimal or no mechanical ventilation during VA ECMO support is sometimes employed to prevent ventilation-induced lung injury (30), although optimal practices are yet to be defined. Although, survivors in our study had a significantly higher minute ventilation of 7 vs. 5 L, that did not independently influence survival. Clinicians should, however, ensure lung protective ventilatory strategies for native lung ventilation in these patients (31).

The strength of this study is that it evaluated ventilation and blood gas parameters during the entire duration of ECMO support to analyse cumulative oxygen exposure. Limitations of this study include it being a single center study involving small cohort of patients. Although we examined many variables, potential unmeasured confounders likely still exist. Importantly, this is a retrospective study and associations observed do not mean causation. Larger prospective studies are required to answer these questions fundamental to VA ECMO practice. Also moving forward, end organ injury attributable to hyperoxaemia may be an important outcome to measure in such studies. This was challenging to measure within the small sample in this study given that most patients exhibited some end organ injury and it is difficult to correlate degrees of hyperoxaemia with degrees of organ injury.

## Conclusion

This exploratory analysis in a small cohort of VA ECMO supported patients demonstrated that hyperoxemia was common during VA ECMO support but was not independently associated with increased in-hospital mortality. Similarly, hospital survivors also received significantly lower EBFRs and higher native lung ventilation, yet this too was not independently associated with mortality. These findings highlight that interactions between EBFR, PaO_2_, and minute ventilation may be more relevant than their individual association with survival. Further research is indicated to determine the optimal EBFR, Fdo_2_, and ventilator settings during VA ECMO.

## Data Availability Statement

The original contributions presented in the study are included in the article/supplementary material, further inquiries can be directed to the corresponding author/s.

## Ethics Statement

The studies involving human participants were reviewed and approved by the Prince Charles Hospital Ethics Committee. Written informed consent for participation was not required for this study in accordance with the national legislation and the institutional requirements.

## Author Contributions

KS conceived the study, contributed significantly to subsequent drafts, and editing of the manuscript. AJ and GC collected the data. CA analyzed the data. AJ wrote the first draft of the manuscript. AB and DB critically evaluated the manuscript and contributed to writing. All authors contributed to the article and approved the submitted version.

## Conflict of Interest

DB reports receiving research support from ALung Technologies. He has been on the medical advisory boards for Abiomed, Xenios, Medtronic, Cellenkos and Hemovent. The remaining authors declare that the research was conducted in the absence of any commercial or financial relationships that could be construed as a potential conflict of interest.

## Publisher's Note

All claims expressed in this article are solely those of the authors and do not necessarily represent those of their affiliated organizations, or those of the publisher, the editors and the reviewers. Any product that may be evaluated in this article, or claim that may be made by its manufacturer, is not guaranteed or endorsed by the publisher.
